# Epigenome-wide association study of depression symptomatology in elderly monozygotic twins

**DOI:** 10.1038/s41398-019-0548-9

**Published:** 2019-09-02

**Authors:** A. Starnawska, Q. Tan, M. Soerensen, M. McGue, O. Mors, A. D. Børglum, K. Christensen, M. Nyegaard, L. Christiansen

**Affiliations:** 1 Department of Biomedicine, Aarhus University, Aarhus, Denmark; 2 The Lundbeck Foundation Initiative for Integrative Psychiatric Research, iPSYCH, Aarhus, Denmark; 3 Center for Integrative Sequencing, iSEQ, Aarhus Genome Centre, Aarhus University, Aarhus, Denmark; 4 The Danish Twin Registry, Institute of Public Health, University of Southern Denmark, Odense, Denmark; 5 Department of Clinical Genetics, Odense University Hospital, Odense, Denmark; 6 The Danish Aging Research Center, Institute of Public Health, University of Southern Denmark, Odense, Denmark; 7 Department of Psychology, University of Minnesota, Minneapolis, MN USA; 8 Psychosis Research Unit, Aarhus University Hospital, Risskov, Denmark; 9 Department of Clinical Biochemistry and Pharmacology, Odense University Hospital, Odense, Denmark; 10 Department of Clinical Immunology, Copenhagen University Hospital, Rigshospitalet, Copenhagen, Denmark

**Keywords:** Genomics, Pathogenesis

## Abstract

Depression is a severe and debilitating mental disorder diagnosed by evaluation of affective, cognitive and physical depression symptoms. Severity of these symptoms strongly impacts individual’s quality of life and is influenced by a combination of genetic and environmental factors. One of the molecular mechanisms allowing for an interplay between these factors is DNA methylation, an epigenetic modification playing a pivotal role in regulation of brain functioning across lifespan. The aim of this study was to investigate if there are DNA methylation signatures associated with depression symptomatology in order to identify molecular mechanisms contributing to pathophysiology of depression. We performed an epigenome-wide association study (EWAS) of continuous depression symptomatology score measured in a cohort of 724 monozygotic Danish twins (346 males, 378 females). Through EWAS analyses adjusted for sex, age, flow-cytometry based blood cell composition, and twin relatedness structure in the data we identified depression symptomatology score to be associated with blood DNA methylation levels in promoter regions of neuropsin (*KLK8*, *p*-value = 4.7 × 10^−7^) and DAZ associated protein 2 (*DAZAP2*, *p*-value = 3.13 × 10^−8^) genes. Other top associated probes were located in gene bodies of *MAD1L1* (*p*-value = 5.16 × 10^−6^), *SLC29A2* (*p*-value = 6.15 × 10^−6^) and *AKT1* (*p*-value = 4.47 × 10^−6^), all genes associated before with development of depression. Additionally, the following three measures (a) DNAmAge (calculated with Horvath and Hannum epigenetic clock estimators) adjusted for chronological age, (b) difference between DNAmAge and chronological age, and (c) DNAmAge acceleration were not associated with depression symptomatology score in our cohort. In conclusion, our data suggests that depression symptomatology score is associated with DNA methylation levels of genes implicated in response to stress, depressive-like behaviors, and recurrent depression in patients, but not with global DNA methylation changes across the genome.

## Introduction

Depression is a multifactorial common psychiatric disorder diagnosed by evaluation of various depressive symptoms, such as low mood, loss of interest and pleasure, fatigue and loss of energy, decline in cognitive functioning, poor concentration, increase in anxiety, inappropriate guilt, change in appetite, sleep disturbance, as well as changes in psychomotor activity^1^. Early diagnosis and treatment of depression is beneficial for the patient and individual’s later mental health outcome^2^. However, due to the complexity of depression symptomatology and its varying severity across the general population, depression is reported to be under-diagnosed and therefore under-treated in society^3–5^. Depression is estimated to be the leading global cause of years lost due to disability worldwide, with lifetime prevalence of the disorder estimated to be ~14%, and even reaching 21% in high-income countries^6,7^. Risk of suffering from depression is influenced by common genetic variants^8–11^, with twin studies attributing ~40% of the variation in depression liability to the additive genetic effects^12,13^, and change in depression symptomatology reported to be a heritable trait, with heritability estimates reaching 30%^14^. Apart from genetic factors, which contribute to but do not fully explain individual’s disorder risk, environmental exposures, such as social and socioeconomic factors (social isolation^15^, life events^16^, low income or financial problems^17^, level of education^18,19^), as well as lifestyle factors (such as diet^20^ or level of physical activity^21^) impact severity of depression symptomatology and risk of developing depression across the lifespan^22^.

One of the molecular mechanisms through which environmental factors can modulate phenotype outcome is epigenetics, with DNA methylation being one of the most studied modifications of the genome. DNA methylation is dynamic and changes across an individual’s lifespan, influenced by prenatal environmental factors^23–26^, life events^27–30^, lifestyle choices^23,31–37^, as well as puberty^38,39^ and aging^40,41^. DNA methylation plays a pivotal role in regulation of human brain development, its functioning, and aberrant changes in this modification are increasingly reported to be associated with mental disorders (schizophrenia, bipolar disorder, mental retardation, ADHD, autism)^42–52^, mental disorder trajectories^53^, and cognitive^54–56^, as well as social functioning^57^.

In this study we hypothesized that there are DNA methylation signatures in the genome that associate with depression symptomatology in the general population. As liability to depression in part constitutes the extreme of a quantitatively measurable depression symptomatology identification of such signatures could inform on genes and molecular pathways involved in progression of depression symptoms, and could allow in the future for earlier identification of individuals at risk of developing depression in the general population. Since both depression symptomatology and DNA methylation are not independent of individual’s genetic background we performed our epigenome-wide association study (EWAS) in a large cohort of Danish monozygotic twins, which allowed us to adjust the analyses for shared genetic and environmental factors.

## Materials and methods

### Study population

The study was performed on a sample of 724 Danish monozygotic twins (378 females and 346 males, representing 362 complete twin pairs) recruited as part of the Danish Twin Registry (DTR)^58,59^ for whom DNA methylation data and depression symptomatology score was available. Participants were enrolled in the survey as a part of the Middle Aged Danish Twin Study (MADT)^60^ and the Longitudinal Study of Aging in Danish Twins (LSADT)^61^, both designed, organized and performed by DTR. Depression symptomatology was assessed for all participating twins by using a nine-symptom ‘affect scale’, corresponding to the affective depression assessment, adapted from the Depression Section of the Cambridge Mental Disorders of the Elderly Examination (CAMDEX), as previously described^62^. The nine questions evaluate the current emotional state of study participants and a final affective depression symptomatology score was calculated as the sum of the nine items. Higher affective depression symptomatology score corresponds to more severe symptoms^14,62^. During the visit whole blood samples were collected for all study participants. All study participants gave informed consent. Permissions to collect blood samples and the usage of register-based information were granted by Regional Committees on Health Research Ethics for Southern Denmark (S-VF-19980072 and S-VF-20040241). Genomic DNA was extracted from buffy-coat fraction with the use of the semi-automated salt precipitation protocol with Autopure System (Qiagen, Hilden, Germany).

### DNA methylation profiling

In total 500 ng of genomic DNA extracted from buffy coat from each individual was bisulfite converted with the use of EZ Methylation Gold Kit (Zymo Research, Irvine, California, United States). Bisulfite converted DNA was further analyzed using the Infinium Human Methylation 450 K array (Illumina, San Diego, California, United States) according to manufacturer’s protocol. Quality control of DNA methylation data was performed with a combination of MethylAid^63^ and minfi^64^ tools. In short probes with high detection *p*-value (>0.01), low bead count (<3 beads), zero signal, missing in >5% of samples and cross-reactive probes, as reported before^65^, were removed from the dataset. In order to reduce the technical variation methylome data was normalized with the use of functional normalization (FunNorm)^66^, which regresses out the technical variability estimated from control probes included in Infinium HumanMethylation450BeadChip. Obtained normalized beta-values were further logit transformed to obtain M-values, as recommended before by Du P. and co-authors^67^. According to the current Danish legislation transfer and sharing of individual-level data requires prior approval from the Danish Data Protection Agency and requires that data sharing requests are delt with on a case-by-case basis. To comply with the study’s ethical approval the data cannot be deposited in a public database, however, we welcome any enquiries regarding collaboration and individual requests for data sharing.

### Blood cell composition

Blood cell counts were available for 477 individuals, where five blood leukocyte subtypes (monocytes, lymphocytes, basophils, neutrophils, eosinophiles) were measured using a Coulter LH 750 Hematology Analyzer (Beckman Coulter, Brea, California, United States). For the remaining individuals, where blood cell composition was not available, data was imputed by partial least squares regression with the use of wbccPredictor tool (https://github.com/mvaniterson/wbccPredictor), as described before^55^. In short, a regression model was first fitted based on the 477 samples for which measurements were available, and afterwards it was applied for prediction of missing cell counts. The model used log(cell count + 1) as response and included all beta values, that were available for all samples, as covariates. Sex, age, and sentrix position were also included as covariates in the model. The sentrix position was modeled by two categorical variables indicating the position in each of the two directions on the chip: column-wise and row-wise. All calculations were performed in R^68^.

### Association of depression symptomatology score with covariates

We performed several exploratory analyses to investigate if measured affective depression symptomatology score is associated with age, sex, and blood cell counts in this cohort of MADT and LSADT monozygotic twins. The analyses were performed with the use of linear mixed models with depression symptomatology score as the outcome variable, age, sex, and blood cell composition as fixed effects, and twin pairing information as a random effect. The analyses were performed with the use of lmerTest^69^ R package.

### Epigenome-wide association studies

In this study we examined if DNA methylation in whole blood is associated with depression symptomatology in middle-aged as well as elderly individuals, while adjusting for their genetic background with a monozygotic twin study design. Possible associations between DNA methylation levels and depression symptomatology score were investigated in two statistical models, first where we studied monozygotic twin intra-pair differences (*paired analysis*), and second model where all individuals were treated as singletons while adjusting for the relatedness structure in the dataset (*unpaired analysis*). For the *paired* analysis differences in the depression symptomatology score, DNA methylation and blood cell composition between twins were calculated for all twin pairs. DNA methylation differences were regressed on depression symptomatology score differences and were adjusted for age, sex and intra-pair differences in blood cell composition. The reason for including sex and age variables in the *paired model* was to adjust for sex and age effects on the intra-pair differences. This aspect is important as the intra-pair differences can increase with increasing age and may also differ between male and female twin pairs. For the *unpaired* regression analysis we used linear mixed models and adjusted for age, sex, individuals’ blood cell composition, and specified twin pairing as a random effect. All analyses were performed in R^68^. All probes with suggestive association *p*-value < 1×10^−5^ were annotated to gene symbols according to human genome assembly (hg19) to provide better overview of the most associated sites in the study. Identified genes were further used for Gene Ontology, KEGG and DisGeNET (human disease) pathway overrepresentation enrichment analysis (ORA) with the use of WebGestalt tool^70^ versus genes included at Infinium HumanMethylation450BeadChip as background.

We also performed a replication of the most associated sites (*p*-value < 1 × 10^−5^) identified in a recent EWAS meta-analysis study of depression symptomatology^71^ in our cohort of Danish monozygotic twins in results obtained from both *paired* and *unpaired* models. We performed replication for all results, from both discovery and meta-analysis, from Story et al. (2018) report^71^.

### Differentially methylated regions

In order to expand the search of epigenetic signatures associated with depression symptomatology we extended our analyses to differentially methylated regions (DMRs) for both *paired* and *unpaired* statistical approaches. DMRs were identified with a comb-p tool^72^ reported to have consistently the best sensitivity and high control of false-positive rate when compared to other DMR tools (DMRcate, bumphunter, and probe lasso)^73^. Comb-p analyses were ran using Python 2.7 with parameters reported to achieve the best performance, as tested for DNA methylation array studies, seed <0.05 and dist = 750^73^. Identified DMRs, consisting of at least 3 probes and reaching unadjusted DMR p-value <0.05, were annotated to gene symbols according to human genome assembly (hg19). Additionally, *p*-value for each DMR was adjusted for multiple testing with Šidák correction method^74^ as implemented by default in the comb-p tool^72^.

### DNA methylation age

Another approach to analyze epigenetic signatures of a studied trait is to investigate associations between DNA methylation age (DNAmAge) and the phenotype of interest. DNAmAge, known also as the epigenetic clock, represents age-related changes in DNA methylation at multiple sites in the genome and provides an alternative to performing single-site analysis as in the case of EWAS approach. DNAmAge was estimated for each individual with Horvath^75^ and Hannum^76^ biological clock estimators, both appropriate for methylome data obtained from blood tissue collected from adult individuals. Horvath and Hannum DNAmAge estimates were further correlated with affective depression symptomatology score with and without adjusting for chronological age of each individual. Additionally, we investigated if deltaDNAmAge (difference between DNAmAge and chronological age of each individual) and accelDNAmAge (residuals from DNAmAge regressed on chronological age of each individual) are associated with depression symptomatology score. The difference between deltaDNAmAge and accelDNAmAge is that the first measure represents age difference at individual level, while the latter one measures an alteration of aging of an individual when compared to the rest of the cohort^56^. All DNAmAge-related depression symptomatology score analyses were performed for both Horvath and Hannum DNAmAge estimates. Analyses were performed with linear mixed models, and were adjusted for sex as fixed effect and specified twin pairing as random effect.

## Results

DNA methylation data for all 724 monozygotic twins included in this study passed all quality control steps performed with minfi and MethylAid pipelines. Overview of demographics of MADT and LSADT twins participating in this study is provided in Table 1. We observed significant associations between depression symptomatology score with sex and with chronological age (see Table 2). Depression symptomatology score was increased in females in comparison to males, and additionally increased with age (see Table 2). We also investigated if blood cell composition is associated with the depression symptomatology score. Out of five studied blood cell types we observed two (lymphocyte and neutrophil proportions) to be significantly associated with the score (see Table 2). Interestingly, levels of associations for these two cell types with depression symptomatology score were very comparable (in terms of absolute effect size and significance level), and the reason for this similarity was a high negative correlation between lymphocyte and neutrophil proportions (*r* = −0.93).

**Table 1:** Demographics of monozygotic twins from MADT and LSADT cohorts included in this study

	MADT (*n* = 486)		LSADT (*n* = 238)	
	Males (*n* = 264)	Females (*n* = 222)	Males (*n* = 82)	Females (*n* = 156)
Age ± sd [years]	66.2 ± 6.1	65.6 ± 6	78.0 ± 4.0	77.9 ± 4.1
Age min/max [years]	56–79	55–79	73–87	73–89
Mean depression score ± sd	10.4 ± 1.8	11.0 ± 2.4	10.9 ± 2.6	11.3 ± 2.7
Depression score min/max	9–20	9–22	9–20	9–22

**Table 2:** Results from association analyses between depression symptomatology score, chronological age, sex, and blood cell composition adjusted for relatedness structure in the data

	Depression symptomatology score	
	Estimate ± sd	*P*-value
Chronological age adjusted for sex	0.03 ± 0.01	**0.01**
Sex adjusted for chronological age	−0.56 ± 0.02	**0.004**
Basophil proportions	−7.2 ± 21.84	0.74
Eosinophil proportions	−9.8 ± 6.50	0.13
Lymphocyte proportions	−3.22 ± 1.14	**0.005**
Neutrophil proportions	3.19 ± 1.06	**0.003**
Monocyte proportions	−1.2 ± 3.14	0.70

**Fig. 1:**
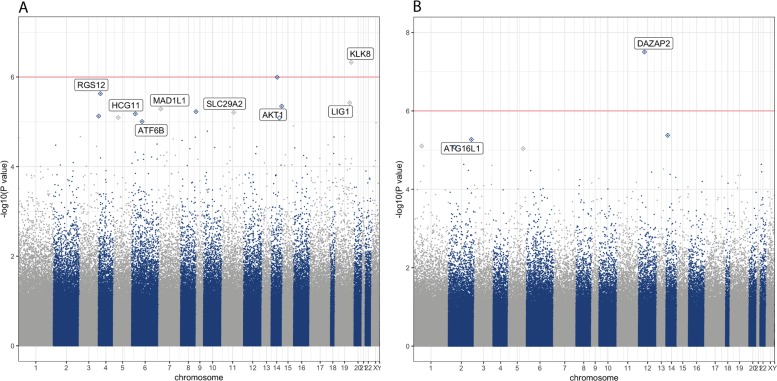
Manhattan plots of depression symptomatology EWAS results from *paired* A and *unpaired* B models adjusted for sex, age and blood cell composition. All loci with *p*-value < 10^−5^ are annotated to genes according to human genome assembly (hg19)

Next, we performed EWAS analyses of depression symptomatology score with both *paired* and *unpaired* twin models. In the *paired* analysis, where we investigated if within-twin-pair depression symptomatology score differences can be associated with within-twin-pair DNA methylation level differences, we identified cg05777061 probe as the most associated finding (*p*-value = 4.7 × 10^−7^, FDR *p*-value = 0.21, see Table 3). The probe is located on chromosome 19 and targets promoter region of kallikrein-8 (*KLK8*) gene, also known as neuropsin gene. The finding in *KLK8* gene was the only one in the *paired* model analysis with *p*-value < 10^−6^, as depicted by a Manhattan plot (Fig. 1a). In the EWAS of depression symptomatology score performed with the *unpaired* approach, where all individuals were treated as singletons and we adjusted for the relatedness structure in the dataset, we identified cg00554948 probe as the most significant finding (*p*-value = 3.13 × 10^−8^, FDR *p*-value = 0.014). The probe is located on chromosome 12 in a promoter region of DAZ Associated Protein 2 (*DAZAP2*) gene, also known as Proline-Rich Transcript In Brain. This was the only finding with association *p*-value < 10^−6^, as depicted by a Manhattan plot (Fig. 1b). We identified suggestive associations (p-value < 10^−5^) for additional 12 loci from the *paired* model targeting *MAD1L1*, *SLC29A2*, *AKT1*, *ATF6B*, *RGS12*, *LIG1*, *HCG11* genes, as well as intergenic regions. For the *unpaired* model we identified additional 5 loci with suggestive association *p*-value < 10^−5^, most of them located in intergenic regions and one located in *ATG16L1*. All differentially methylated sites with suggestive association *p*-value < 10^−5^ are presented in Table 3. Inflation factor λ was calculated for of each of the performed EWAS analysis with the use of ramwas R package^77^. λ was estimated to be 1.014 for the *paired* EWAS, and 0.92 for the *unpaired* EWAS indicating little deflation or inflation of our EWAS results from the expected distribution of p-values.

**Table 3:** The most associated probes (*p*-value < 10^−5^) from *paired* and *unpaired* EWAS analyses of depression symptomatology score adjusted for sex, age, and blood cell composition

Probe ID	Estimate	se	*P*-value	Probe position (hg19)	Gene	Genomic feature
*Paired twin model*						
cg01859717	−0.027	0.006	9.87E-06	chr6: 32088654	*ATF6B*	Body
cg01919885	0.029	0.006	2.36E-06	chr4: 3365330	*RGS12*	Body, CGI^a^
cg02286193	0.031	0.006	1.01E-06	chr14: 76823128	NA	IGR^b^, CGI^a^ shelf
**cg05777061**	**−0.057**	**0.011**	**4.7E-07**	**chr19: 51505001**	***KLK8***	**TSS200** ^**c**^ **, CGI** ^a^ **shore**
cg10100767	0.030	0.006	4.47E-06	chr14: 105246561	*AKT1*	Body
cg10778249	−0.021	0.004	3.75E-06	chr19: 48674746	*LIG1*	TSS1500^d^, CGI^a^ shore
cg12836280	−0.031	0.007	8.01E-06	chr5: 50260240	NA	IGR^b^
cg15022049	−0.021	0.005	6.15E-06	chr11: 66137145	*SLC29A2*	Body, CGI^b^ shore
cg16135936	0.035	0.008	8.18E-06	chr14: 98629292	NA	IGR^b^
cg17350432	−0.016	0.003	7.47E-06	chr4: 841569	NA	IGR^b^, CGI^a^ shore
cg20250722	−0.018	0.004	6.64E-06	chr6: 26522136	*HCG11*	Body, CGI^a^
cg20556803	0.030	0.006	5.16E-06	chr7: 2114593	*MAD1L1*	Body, CGI^a^ shore
cg26241863	−0.028	0.006	5.93E-06	chr8: 145849419	NA	IGR, CGI^a^ shore
*Unpaired twin model*						
**cg00554948**	**−0.016**	**0.003**	**3.13E-08**	**chr12: 51631858**	***DAZAP2***	**TSS1500** ^**d**^ **, CGI** ^a^ **shore**
cg01971269	−0.008	0.002	9.1E-06	chr5: 162993061	NA	IGR^b^
cg03550773	0.011	0.002	4.2E-06	chr14: 35163458	NA	IGR^b^
cg23050873	−0.009	0.002	5.4E-06	chr2: 234184376	*ATG16L1*	Body
cg25104234	−0.014	0.003	8.4E-06	chr2: 52281777	NA	IGR^b^
cg26603050	0.016	0.004	7.9E-06	chr1: 22938172	NA	IGR^b^

Probes with p-value < 10^−6^ are indicated in bold

^a^CGI: CpG Island

^b^IGR: Intergenic Region

^c^TSS200: Probe positioned within 200 bp region from transcription start site

^d^TSS1500: Probe positioned within 1500 bp region from transcription start site

Top enrichment results for ORA pathway analysis from *paired* EWAS of depression symptomatology were: ‘Regulation of Myelination’ (*p*-value = 8.78 × 10^−5^) for Gene Ontology, ‘Longevity Regulating pathway’ (*p*-value = 9.43 × 10^−4^) for KEGG, ‘Schizophrenia’ (*p*-value = 1.34 × 10^−3^) for DisGeNET analysis (Supplementary Table 1). None of the pathways remained significant after FDR *p*-value correction. ORA performed on genes reported in the *unpaired* EWAS of depression symptomatology did not identify any significantly enriched pathways.

Replication of the most associated sites from a recent EWAS meta-analysis study of depression symptomatology by Story et al.^71^ in our cohort replicated cg07012687 in *SLC16A3* (*p*-value_twin cohort _= 8.75 × 10^−4^, *p*-value_Story-discovery _= 3.47 × 10^−7^, *p*-value_Story-replication _= 1.58 × 10^−1^, *p*-value _Story-meta-analysis _= 4.45 × 10^−6^) as the most associated finding. The second most associated finding within the 51 probes investigated for replication was cg12764201 in *CORT* (*p*-value_twin cohort _= 3.01 × 10^−3^, *p*-value_Story-discovery _= 7.15 × 10^−6^, *p*-value_Story-replication _= 7.20 × 10^−1^, *p*-value_Story-meta-analysis _= 7.29 × 10^−5^). Probe cg04987734 in *CDC42BPB*, identified as one of three genome-wide significant findings in the meta-analysis^71^, replicated as third most significant finding across the investigated 51 probes (*p*-value_twin cohort _= 8.64 × 10^−3^, *p*-value_Story-discovery _= 4.93 × 10^−8^, *p*-value_Story-replication _= 4.82 × 10^−2^, *p*-value_Story-meta-analysis_ = 1.57 × 10^−8^). In total 12 of the investigated 51 probes reached nominal *p*-value < 0.05 in either *paired* or *unpaired* twin analysis. Our most significant findings in *KLK8* and *DAZAP2* were not reported to have *p*-value < 10^−5^ in the Story et al. report^71^. Overview of replication results in our Danish monozygotic twin cohort for all 51 investigated probes from Story et al.^71^ is presented in Supplementary Table 2.

Results from *paired* and *unpaired* EWAS of depression symptomatology obtained from MADT and LSADT monozygotic twin cohorts were further used to identify possible DMRs across the genome associated with this trait. We identified 30 DMRs from *paired* analysis and 40 DMRs from the *unpaired* analysis to be associated with depression symptomatology score with unadjusted DMR *p*-value < 0.05. Overview of all DMRs and their annotated genes is presented in Table 4. Five genes overlapped between DMR *paired* and *unpaired* analysis (*PCDHGA4*, *GLIPR1L2*, *STAM*, *VARS2*, *MAST3*), 2 genes overlapped between *paired* EWAS and *paired* DMR analysis (*MAD1L1* and *RGS12*), and 1 gene overlapped between *paired* EWAS and *unpaired* DMR analysis (*ATF6B*). Overview of all probes located within each of the identified DMRs is presented in Supplementary Table 3 (*paired*) and Supplementary Table 4 (*unpaired*).

**Table 4:** Overview of all DMRs associated with depression symptomatology (DMR unadjusted *p*-value < 0.05) identified with comb-p tool

DMR position (hg19)	Number of probes in the DMR	Stouffer-Lipta p-value for DMR	DMR *p*-value after Šidák adjustment for multiple testing	Gene
DMRs from *paired* analysis				
chr12: 75784616-75785295	10	4.26E-16	2.97E-13	*GLIPR1L2*
chr10: 17685928-17686414	8	2.12E-13	1.98E-10	*STAM*
chr1: 205819087-205819609	7	3.11E-13	2.70E-10	*PM20D1*
chr6: 31650734-31651278	18	2.72E-12	2.27E-09	NA
chr6: 31734105-31734580	12	1.45E-11	1.38E-08	*C6orf27*
chr1: 1067098-1067223	3	4.66E-09	1.69E-05	NA
chr6: 30228046-30228254	10	1.24E-08	2.71E-05	*HLA-L*
chr2: 241076281-241076441	6	5.67E-08	1.61E-04	*MYEOV2*
chr6: 32223075-32223236	7	2.19E-07	6.18E-04	NA
chr12: 132663674-132663883	4	3.46E-07	7.51E-04	NA
chr4: 3432341-3432546	3	4.59E-07	1.02E-03	*RGS12*
chr6: 30882640-30882708	4	5.05E-07	3.36E-03	*VARS2*
chr16: 85253979-85254209	3	7.24E-07	1.43E-03	NA
chr22: 22901568-22901697	5	1.56E-06	5.46E-03	*PRAME, LOC648691*
chr12: 12848976-12849269	8	1.73E-06	2.67E-03	*GPR19*
chr19: 18234710-18234911	3	1.87E-06	4.21E-03	*MAST3*
chr7: 1952517-1952600	3	1.88E-06	1.02E-02	*MAD1L1*
chr5: 2537495-2537834	6	2.98E-06	3.97E-03	NA
chr19: 28284490-28284741	3	3.59E-06	6.46E-03	*LOC148189*
chr5: 140792510-140792700	5	3.85E-06	9.15E-03	*PCDHGA4, PCDHGA6*
chr3: 10806021-10806288	5	4.42E-06	7.47E-03	*LOC285370*
chr19: 2294886-2295092	3	5.58E-06	1.22E-02	*LINGO3*
chr4: 24796987-24797176	5	8.48E-06	2.01E-02	*SOD3*
chr4: 3516533-3516758	4	8.58E-06	1.71E-02	*LRPAP1*
chr1: 234871409-234871477	3	9.47E-06	6.12E-02	NA
chr10: 5406889-5407119	8	9.57E-06	1.87E-02	*UCN3*
chr17: 77916732-77916892	3	1.09E-05	3.04E-02	*TBC1D16*
chr8: 144896175-144896307	3	1.14E-05	3.85E-02	*SCRIB, MIR937*
chr10: 1975561-1975631	3	1.59E-05	9.77E-02	NA
chr19: 46999054-46999118	3	4.25E-04	9.51E-01	*PNMAL2*
DMRs from *unpaired* analysis				
chr11: 67417957-67418405	13	1.87E-14	1.90E-11	*ACY3*
chr6: 31650734-31651291	20	5.78E-14	4.70E-11	NA
chr12: 75784616-75785295	10	8.15E-13	5.44E-10	*GLIPR1L2*
chr6: 31762352-31762776	14	2.16E-11	2.31E-08	*VARS*
chr10: 17685697-17686414	10	4.68E-11	2.96E-08	*STAM*
chr6: 33048253-33048919	23	5.56E-11	3.78E-08	*HLA-DPB1*
chr6: 30882640-30883203	9	2.28E-10	1.83E-07	*VARS2*
chr19: 2428349-2428677	4	3.29E-09	4.55E-06	*LMNB2, TIMM13*
chr6: 117802587-117802786	5	4.92E-08	1.12E-04	*DCBLD1*
chr1: 26233375-26233709	10	1.10E-07	1.50E-04	*STMN1*
chr13: 110521955-110522297	5	1.14E-07	1.50E-04	NA
chr6: 32552015-32552205	6	8.77E-07	2.09E-03	*HLA-DRB1*
chr7: 24323674-24323939	7	9.23E-07	1.58E-03	*NPY*
chr10: 104196205-104196339	4	1.18E-06	3.98E-03	*MIR146B*
chr6: 34206399-34206683	4	1.35E-06	2.16E-03	*HMGA1*
chr6: 32223075-32223341	9	1.76E-06	2.99E-03	NA
chr19: 18234710-18234911	3	2.22E-06	5.00E-03	*MAST3*
chr14: 91818496-91818668	3	2.37E-06	6.22E-03	*CCDC88C*
chr17: 79495267-79495519	6	2.43E-06	4.36E-03	*FSCN2*
chr3: 45635930-45636386	7	2.54E-06	2.52E-03	*LIMD1*
chr10: 134150450-134150690	7	3.17E-06	5.98E-03	*LRRC27*
chr14: 31343282-31343427	3	3.90E-06	1.21E-02	*COCH*
chr12: 10183166-10183364	7	4.01E-06	9.13E-03	*CLEC9A*
chr12: 7260545-7260776	6	4.23E-06	8.27E-03	*C1RL, LOC283314*
chr11: 62621177-62621406	4	5.02E-06	9.88E-03	*SNORD30, SNORD22*
chr9: 4662857-4663107	3	6.13E-06	1.11E-02	*C9orf68, PPAPDC2*
chr4: 74847645-74847829	7	7.31E-06	1.79E-02	*PF4*
chr1: 244094868-244094935	3	8.53E-06	5.61E-02	NA
chr2: 71211980-71212157	3	1.06E-05	2.67E-02	*ANKRD53*
chr11: 57408513-57408751	3	1.43E-05	2.68E-02	*MIR130A*
chr6: 32086754-32086928	10	1.80E-05	4.58E-02	*ATF6B*
chr10: 48416780-48416977	7	2.20E-05	4.94E-02	*GDF2*
chr7: 3227261-3227332	3	2.29E-05	1.36E-01	NA
chrX: 114524263-114524470	6	2.90E-05	6.15E-02	*LUZP4*
chr10: 74034643-74034667	3	2.93E-05	4.25E-01	*DDIT4*
chr17: 8127195-8127373	3	3.06E-05	7.49E-02	*C17orf44*
chr6: 29911541-29911558	3	3.82E-04	1.00E + 00	*HLA-A*
chr11: 117069848-117069966	5	8.26E-04	9.58E-01	*TAGLN*
chr5: 140792595-140792700	3	1.24E-02	1.00E + 00	*PCDHGA4, PCDHGA6*
chr5: 92956643-92956679	3	3.78E-02	1.00E + 00	*FAM172A, MIR2277*

Apart from studying epigenetic signatures of depression symptomatology score in a single-CpG-site resolution manner we also investigated if blood-derived DNAmAge is associated with the depression symptomatology score. We observed a high Pearson’s correlation of both Horvath (*r* = 0.80) and Hannum (*r* = 0.79) DNAmAge estimates with chronological age of study participants. Horvath DNAmAge measure underestimated (mean = 65.07 years ± 9.49, min = 43.35 years, max = 107.96 years), while Hannum DNAmAge measure overestimated individuals’ age (mean = 74.30 years ± 8.31, min = 54.85 years, max = 108.75 years) in comparison to chronological age of the twin cohort (mean = 69.89 years ± 7.86, min = 55 years, max = 89 years). Both Horvath and Hannum DNAmAge estimates were found to be significantly associated with depression symptomatology score (*p*-values < 0.05), however, these findings did not remain significant after adjusting the models for chronological age (*p*-values >0.05). This result was also reflected in further regression analyses where no association was observed between depression symptomatology score and deltaDNAmAge and accelDNAmAge (see Table 5), as both represent measures from which chronological age was either subtracted or regressed. Overview of all results from association analyses performed between depression symptomatology score with Horvath and Hannum DNAmAge estimates adjusted for sex and relatedness structure in the data is presented in Table 5.

**Table 5:** Results from association analyses between depression symptomatology score and DNA methylation age estimates adjusted for sex and relatedness structure in the data

Regression model	Depression symptomatology score *p*-value
DNAmAge Horvath	
Unadjusted for chronological age	**0.039**
Adjusted for chronological age	0.88
DeltaDNAmAge	0.95
AccelDNAmAge	0.86
DNAmAge Hannum	
Unadjusted for chronological age	**0.026**
Adjusted for chronological age	0.71
DeltaDNAmAge	0.88
AccelDNAmAge	0.66

## Discussion

Depression is a complex mood disorder influenced by a combination of genetic and environmental factors. One of the molecular mechanisms that allows for an interplay between genes and environment is epigenetics, which through its dynamic nature has the potential of continuously contributing to the pathophysiology of depressive symptoms. In this study we performed an EWAS of depression symptomatology in a large cohort of monozygotic twins with two different statistical approaches and we identified DNA methylation levels in *KLK8* and *DAZAP2* genes to be most associated with the depression symptomatology score. The two EWAS data analysis approaches (*paired* and *unpaired*) differ in how they estimate and handle confounding factors, as described before^55^. In short the first model analyzes intra-twin-pair differences which are adjusted for the effects from pair-specific confounding factors (age, sex, genetics, shared environmental factors), while the second model, even though also corrects for age and sex, does that for the effects on individual DNA methylation levels and also allows for inclusion of incomplete twin pairs if such are present^55^.

*KLK8*, also known as neuropsin, encodes a serine protease and maps to chromosome 19q13, a region implicated in schizophrenia and bipolar disorder by genetic linkage studies^78,79^. Genetic variation in human neuropsin itself was associated in a candidate-gene study with bipolar disorder and cognitive functioning, however not with major depression^80^. Neuropsin exists in two forms: a regular neuropsin (type 1) and hominoid-specific neuropsin (type 2)^81^, the latter containing a 135-bp insertion in 5′ upstream region of exon 3. Both types of neuropsin are abundantly expressed in human brain, with type 2 reported to be preferentially expressed in the adult brain, including hippocampus, frontal lobe and cerebral cortex^80–82^. Neuropsin is involved in synaptogenesis, maturation of orphan and small synaptic boutons^83^, and is responsible for degradation of cell adhesion molecule L1 (CAM-L1)^84^. Interestingly, *CAM-L1* levels were found to be increased in prefrontal cortex and decreased in parieto-occipital cortex in post mortem brains of depressed individuals^85^. Exposure of rats to prolonged stress resulted in decreased CAM-L1 brain levels, while antidepressant treatment increased its expression^86^. In the same line acute stress was shown to increase neuropsin mRNA levels in mouse hippocampus^87^, while inactivation of neuropsin was shown to have protective effects against depressive-like behaviors and memory impairment induced by chronic stress in mice^88^. Significant increase in expression levels of human neuropsin, measured in peripheral blood, was observed between patients suffering from recurrent depression episodes compared to patients with first episode of depression^89^. These findings were further supported by a follow-up study where increased mRNA levels of neuropsin were found in patients with recurrent depression in comparison to healthy controls^90^. These studies support our observation of association between differential levels of DNA methylation in the promoter region of neuropsin and depression symptomatology, and further suggest that these changes may be modulated by external factors (such as chronic stress, acute stress or use of antidepressants).

The second probe identified in this study was located in the promoter region of *DAZAP2*, a well-conserved gene known for inducing stress granule formation^91,92^. According to Enrichr database DAZAP2 directly interacts with HGS, NEDD4, UBQLN4, UBB, UBC, MAP3K7, SMURF2, CTNNB1, ATXN1, and RPS27A^93^, most of these genes have been reported before to be associated with major depression, depression symptomatology, as well as exposure to stress^94–101^. Recent methylome analysis of monozygotic twins discordant for childhood psychotic symptoms identified a differentially methylated site located closest to *DAZAP2* among the top ten most associated findings with the phenotype, however the probe was located -19713 bp from *DAZAP2* TSS, while our probe was located closer to the gene, within the -1500 bp region from its TSS^102^. Differential methylation of *DAZAP2* promoter region was shown to regulate its expression in multiple myeloma cell lines^103^, and its decreased expression levels were found to contribute to pathogenesis of this cancer^104^. Therefore, possible link between *DAZAP2* and depression symptomatology requires further investigation. Differences between the top results observed from *paired* and *unpaired* EWAS analyses may be attributed to different statistical approaches that they use to evaluate the associations between DNA methylation levels and the studied trait, therefore interpretation of results from these two models differs. However, it is worth to note the overlap between genes identified from *paired* and *unpaired* DMR analyses, both based on the initial EWAS findings. This overlap indicates that even though these two models are not statistically equal they are both capable of identifying the same epigenetic signatures of depression symptomatology score.

Further investigation of other most differentially methylated sites in the EWAS analyses identified epigenetic changes in additional genes of high interest to the depression phenotype, such as mitotic arrest deficient 1 like 1 (*MAD1L1*), solute carrier family 29 member 2 (*SLC29A2*), AKT serine/threonine kinase 1 (*AKT1*). Recent large genome-wide association studies reported genetic variation in *MAD1L1* to be genome-wide significantly associated with ICD-coded major depressive disorder (MDD)^105^, schizophrenia^106^, and bipolar disorder^107^. Additionally, a study comparing monozygotic twins discordant for MDD reported affected twins to have greater variance in methylation in *MAD1L1* than their unaffected co-twins^108^, supporting our observation of association between depression phenotype and DNA methylation levels of *MAD1L1*. Genetic polymorphism in *SLC29A2* showed suggestive association in candidate-gene studies (*p*-value < 0.05) with depression and depression with fatigue phenotypes in men^109^, as well with childhood trauma score within MDD cases^110^. Genetic polymorphism in *AKT1* was associated with severity of depression, anxiety symptoms and suicide attempts in patients with MDD from a Chinese Han population^111^, with late-onset depression in a Brazilian population^112^, and antidepressant treatment response in patients with depressive disorder in a Caucasian population^113^. Differential methylation of *AKT1* in blood was associated with clinical post-traumatic stress disorder in combat veterans^114^, and in post-mortem brain of schizophrenia patients compared to unaffected controls^115^, while changes in its expression level in blood were positively correlated with improvement of depression symptoms among bipolar disorder patients treated with lithium^116^. Altogether these studies support involvement of genes identified as differentially methylated in this study in the development of depression symptoms.

Other studies that investigated DNA methylation signatures of depression in discordant monozygotic twins study design implicated epigenetic changes in *VDR26*, *HOXB7*, *CACNA1C, STK32C, NR1C3*, and *MYC* genes among others, but not in *KLK8* or *DAZAP2*^47,108,117,118^. These studies were performed mainly in blood samples, but also in buccal cells of monozygotic twins discordant for depression, and even though they were performed on smaller twin cohorts, they studied individuals from a more severe part of depression scale in comparison to the MADT and LSADT twins analyzed in this study. That is why it should be noted that our findings represent epigenetic results related to severity of depression symptomatology across the general population, rather than markers related to MDD diagnosis. We have also observed that apart from the signal from epigenetic associations the depression symptomatology score was increased in females in comparison to males, and increased additionally with age, as reported before for elderly individuals^22^. Depression symptoms were reported to be comparable between younger and elderly individuals suffering from depression^119,120^. However, it should be noted that depression symptomatology evaluated in elderly individuals, in comparison to the young ones in the general population, may be driven by different factors, such as cognitive decline or represent a prodromal feature of dementia^121–123^. Based on the available data in this study we cannot delineate if the observed epigenetic differences associated with depression symptomatology in the elderly are generalizable to younger individuals. Other studies that identified changes in *KLK8* expression levels to be linked with recurrent depression were performed in a younger population aged 18–67 (mean = 47.64 years, sd = 11.16)^89^ than MADT and LSADT cohorts, but more research is required across even younger age groups to assess if findings from this study apply to the general population.

In this study we have also tested the hypothesis that increased individual’s biological age (measured with Horvath- and Hannum-based DNAmAge estimates) in comparison to individual’s chronological age, which may serve as a marker of accelerated aging and health deterioration, associates with more severe depression symptomatology score in elderly individuals. Similarly to previous studies on cohorts with comparable age-spans we observed that Horvath-based DNAmAge underestimated, while Hannum-based DNAmAge overestimated the age of our study participants^40,56^. This difference could be explained by different set of probes used in these two DNAmAge predictors, with only 6 probes out of 353 in Horvath and 71 in Hannum DNAmAge estimators overlapping between them^75,76^. We initially observed a positive association between both Horvath and Hannum DNAmAge estimates and depression symptomatology score, however it was no longer significant after adjusting for chronological age of these individuals, a variable associated with the score itself. This observation suggests that the initial effect was observed only due to high correlation between DNAmAge and chronological age, and further between chronological age and depression symptomatology score. A recent study reported accelerated epigenetic aging in individuals suffering from MDD in comparison to controls^124^. The reason why we did not detect any association between accelDNAmAge and depression symptomatology score in our study may be because the phenotype in our study differed from the one studied by Han and co-authors, as we studied only depression symptomatology measured in a general population of monozygotic twins, and not in patients diagnosed with MDD, who, as pointed out before, represent the most severe part of the depression symptomatology spectrum. Thus, further studies, preferably performed in a cohort of monozygotic twins discordant for MDD, are needed to elucidate if DNAmAge acceleration is associated with the disorder regardless of the genetic background of studied individuals.

Apart from many strengths of this study, such as use of a large cohort of monozygotic twins and adjustment of EWAS models for blood cell proportions measured with flow cytometry, there are also potential limitations of this study, such as use of blood samples to study a brain-related phenotype. We are aware that DNA methylation profiles are tissue-specific, that they substantially differ between brain and blood samples, and that if surrogate tissues should be informative on the molecular changes of tissue of interest their methylation levels at locus of interest should ideally co-vary^125,126^. For the two most associated sites with *p*-value < 10^−6^ (in *KLK8* and *DAZAP2* genes) we did not find any strong evidence for correlation of methylation levels between blood and prefrontal cortex (PFC), entorhinal cortex (EC), superior temporal gyrus and cerebellum regions (https://epigenetics.essex.ac.uk/bloodbrain/)^126^. The most significant correlation for methylation levels for cg05777061 was observed between blood and EC (*p*-value = 0.026, *r* = 0.26) and for cg00554948 between blood and PFC (*p*-value = 0.035, *r* = 0.25). Remaining comparisons for these four tissues for these two probes had *p*-value > 0.05. Interestingly, previous studies did report factors that impact brain methylome to also leave an epigenetic signature in blood, and that inter-individual differences detected in blood correlate with differences observed in brain^125,127^. The latter observation may be explained to some extent by cross-tissues mQTL signals^126,128^, however a recent study reported DNA methylation differences observed in buccal cells between monozygotic twins discordant for depression to be successfully replicated in independent brain samples, which supports the use of secondary tissues for research of mental disorders^117^. That is why further replication of our findings in brain samples collected from an independent monozygotic twin cohort with data on depression symptomatology score is of high interest.

Use of the monozygotic twin study design has many advantages, as it allows adjusting the analyses for various factors shared between analyzed co-twins. However, the results could still be influenced by the unmeasured non-shared environmental factors, such as the well-known confounder of epigenomic studies tobacco smoking. None of our top associated sites was located in the well-established tobacco smoking loci, such as *AHRR* or *GPR15*, and none of the recent tobacco smoking EWAS reported DNA methylation changes in *KLK8* or *DAZAP2* to be associated with this trait^31,129–135^. Therefore we believe that tobacco smoking was not a confounder in our EWAS of depression symptomatology, or at least it was not a strong one. Another possible confounder that could impact our results was use of drugs, especially use of antidepressants is of interest in the context of this phenotype. According to the Drug-Gene Interaction Database neither *KLK8* nor *DAZAP2* have any well-established drug interactions^136^, however, an antidepressant fluoxetine was reported before to alter expression of *KLK8* in the mice hippocampus^137^. Therefore we cannot exclude that the reported results are not influenced by additional non-shared environmental factors between investigated monozygotic twins.

It should be also noted that both top findings reported and discussed in this study (*KLK8* and *DAZAP2*) were identified by using a *p*-value cut-off < 1 × 10^−6^, however, there is a discrepancy in the scientific literature on the threshold for epigenome-wide significant findings. Initially EWAS findings with *p*-value < 1 × 10^−6^ were considered to be genome-wide significant^138^, but a recent study proposed a more stringent threshold with *p*-value < 2.4 × 10^−7^ for the 450 K Illumina methylation array significant findings threshold, and *p*-value < 3.6 × 10^−8^ for genome-wide significant findings to be used^139^. Inclusion of more individuals with higher depression symptomatology score could allow for identification of more significant and stronger signals. These signals could also be informative on the epigenetic changes most related to the diagnosis of MDD. Also it should be remembered that the MADT and LSADT cohorts used in this study were composed only of elderly individuals and that their depression symptomatology score was evaluated with an instrument adequate for this age-span (CAMDEX)^62^. However, whether there are different biological processes that impact depression symptomatology at younger ages is yet to be elucidated.

In conclusion, we have performed EWAS of depression symptomatology score in a unique cohort of elderly monozygotic twins and identified blood methylation levels at *KLK8*, *DAZAP2, MAD1L1, SLC29A2, AKT1*, and other genes, as well as several DMRs across the genome to be associated with this trait. Function of these genes suggests a possible link between exposure to stress, epigenetic regulation of their expression, and further change in depression symptomatology, however, this hypothesis needs to be tested by future studies.

## Supplementary information

Supplementary data legends.

Supplementary Table 1.

Supplementary Table 2.

Supplementary Table 3.

Supplementary Table 4.
