# Supplementary material for: Epigenome-wide association study of depression symptomatology in elderly monozygotic twins
Source: Transl Psychiatry. 2019 Sep 2;9:214. doi: 10.1038/s41398-019-0548-9 (PMC6718679; doi:10.1038/s41398-019-0548-9)
Supplement: Supplementary file 1 — Supplementary data legends. [file 41398_2019_548_MOESM1_ESM.docx]

**Supplementary data legends**

Supplementary Table 1. Overview of results from ORA performed with WebGestalt based on genes annotated to differentially methylated positions with p-value < 10^-5^ from *paired* EWAS analysis of depression symptomatology.

Supplementary Table 2. Replication of the most associated sites from a recent EWAS meta-analysis of depression symptomatology (Story JO et al., 2018) in our cohort of Danish monozygotic twins. Replication overview is presented for all probes that reached p-value <10^-5^ in either discovery (n=7948) or meta-analysis (n=11256). Probes marked in bold indicate loci with nominal p-value < 0.05 in our monozygotic Danish twin cohort (n=724) in either *paired* or *unpaired* association model.

Supplementary Table 3. Overview of all probes located within each of the identified DMRs associated with depression symptomatology score from *paired* analysis.

Supplementary Table 4. Overview of all probes located within each of the identified DMRs associated with depression symptomatology score from *unpaired* analysis.
