# Supplementary material for: Epigenome-wide association study of depression symptomatology in elderly monozygotic twins
Source: Transl Psychiatry. 2019 Sep 2;9:214. doi: 10.1038/s41398-019-0548-9 (PMC6718679; doi:10.1038/s41398-019-0548-9)
Supplement: Supplementary file 2 — Supplementary Table 1. [file 41398_2019_548_MOESM2_ESM.docx]

Supplementary Table 1. Overview of results from ORA performed with WebGestalt based on genes annotated to differentially methylated positions with p-value < 10^-5^ from *paired* EWAS analysis of depression symptomatology.

|  | **Gene Set** | | **Description** | **Size** | **Expect** | **Ratio** | **P-value** | **FDR** |
| --- | --- | --- | --- | --- | --- | --- | --- | --- |
| **Gene Ontology (Biological Processes)** | | GO:0031641 | regulation of myelination | 31 | 0.015 | 137.03 | 0.000088 | 0.78 |
|  | | GO:1903038 | negative regulation of leukocyte cell-cell adhesion | 107 | 0.050 | 39.70 | 0.0011 | 0.94 |
|  | | GO:0042552 | myelination | 111 | 0.052 | 38.27 | 0.0011 | 0.94 |
|  | | GO:0007272 | ensheathment of neurons | 113 | 0.053 | 37.59 | 0.0012 | 0.94 |
|  | | GO:0008366 | axon ensheathment | 113 | 0.053 | 37.59 | 0.0012 | 0.94 |
|  | | GO:0080135 | regulation of cellular response to stress | 603 | 0.284 | 10.57 | 0.0021 | 0.94 |
|  | | GO:0022408 | negative regulation of cell-cell adhesion | 152 | 0.072 | 27.95 | 0.0021 | 0.94 |
|  | | GO:0048585 | negative regulation of response to stimulus | 1429 | 0.673 | 5.95 | 0.0023 | 0.94 |
|  | | GO:0015911 | plasma membrane long-chain fatty acid transport | 5 | 0.002 | 424.80 | 0.0024 | 0.94 |
|  | | GO:0032077 | positive regulation of deoxyribonuclease activity | 5 | 0.002 | 424.80 | 0.0024 | 0.94 |
| **Pathway analysis (KEGG)** | | hsa04211 | Longevity regulating pathway | 87 | 0.051 | 39.32 | 0.00094 | 0.10 |
|  | | hsa04914 | Progesterone-mediated oocyte maturation | 93 | 0.054 | 36.79 | 0.0011 | 0.10 |
|  | | hsa04668 | TNF signaling pathway | 106 | 0.062 | 32.27 | 0.0014 | 0.10 |
|  | | hsa04926 | Relaxin signaling pathway | 129 | 0.075 | 26.52 | 0.0021 | 0.10 |
|  | | hsa04728 | Dopaminergic synapse | 130 | 0.076 | 26.32 | 0.0021 | 0.10 |
|  | | hsa05161 | Hepatitis B | 132 | 0.077 | 25.92 | 0.0022 | 0.10 |
|  | | hsa04915 | Estrogen signaling pathway | 135 | 0.079 | 25.34 | 0.0023 | 0.10 |
|  | | hsa04261 | Adrenergic signaling in cardiomyocytes | 143 | 0.084 | 23.92 | 0.0025 | 0.10 |
|  | | hsa04022 | cGMP-PKG signaling pathway | 158 | 0.092 | 21.65 | 0.0031 | 0.11 |
|  | | hsa05203 | Viral carcinogenesis | 194 | 0.113 | 17.63 | 0.0047 | 0.15 |
| **Disease (DisGeNET)** | | C0036341 | Schizophrenia | 964 | 0.659 | 6.07 | 0.0013 | 0.77 |
|  | | C1858033 | Asymmetric chest | 5 | 0.003 | 292.64 | 0.0034 | 0.77 |
|  | | C0745730 | Multiple lipomata | 5 | 0.003 | 292.64 | 0.0034 | 0.77 |
|  | | C1863351 | Calvarial hyperostosis | 5 | 0.003 | 292.64 | 0.0034 | 0.77 |
|  | | C4280519 | Increased ossification of calvarial bones | 5 | 0.003 | 292.64 | 0.0034 | 0.77 |
|  | | C4280520 | Enlargement of calvarial bones | 5 | 0.003 | 292.64 | 0.0034134 | 0.77 |
|  | | C0162819 | Skin Diseases, Vascular | 5 | 0.003 | 292.64 | 0.0034134 | 0.77 |
|  | | C0334082 | NEVUS, EPIDERMAL (disorder) | 5 | 0.003 | 292.64 | 0.0034134 | 0.77 |
|  | | C0428791 | Aortic valve calcification | 5 | 0.003 | 292.64 | 0.0034134 | 0.77 |
|  | | C0023221 | Leg Length Inequality | 6 | 0.004 | 243.87 | 0.004095 | 0.77 |
