# Supplementary material for: Epigenome-wide association study of depression symptomatology in elderly monozygotic twins
Source: Transl Psychiatry. 2019 Sep 2;9:214. doi: 10.1038/s41398-019-0548-9 (PMC6718679; doi:10.1038/s41398-019-0548-9)
Supplement: Supplementary file 3 — Supplementary Table 2. [file 41398_2019_548_MOESM3_ESM.docx]

Supplementary Table 2. Replication of the most associated sites from a recent EWAS meta-analysis of depression symptomatology (Story JO et al., 2018) in our cohort of Danish monozygotic twins. Replication overview is presented for all probes that reached p-value <10^-5^ in either discovery (n=7948) or meta-analysis (n=11256). Probes marked in bold indicate loci with nominal p-value < 0.05 in our monozygotic Danish twin cohort (n=724) in either *paired* or *unpaired* association model.

| **Probe ID** | **Discovery p-value in Story JO et al., 2018 (n=7948)** | **Replication p-value in Story JO et al., 2018 (n=3308)** | **Meta-analysis p-value in Story JO et al., 2018 (n=11256)** | **P-value paired MZ twin model (n=724)** | **P-value unpaired MZ twin model (n=724)** | **Genomic position (hg19)** | **Gene** | **Genomic feature** |
| --- | --- | --- | --- | --- | --- | --- | --- | --- |
| cg00153395 | >1.00E-05 | >1.00E-05 | 1.02E-06 | 5.29E-01 | 2.70E-01 | chr1:65327523 | *JAK1* | Body |
| cg01541347 | 5.64E-06 | 3.77E-01 | 8.46E-04 | 7.19E-01 | 8.39E-01 | chr7:4729920 | *FOXK1* | Body |
| cg01947751 | 6.23E-06 | 6.63E-01 | 3.68E-04 | 4.07E-01 | 4.76E-01 | chr3:196728969 | NA | IGR^a^, CGI^b^ |
| cg02341197 | 5.84E-06 | 2.02E-01 | 6.80E-06 | 7.78E-01 | 9.24E-01 | chr21:34185927 | *C21orf62* | 5'UTR^d^ |
| cg03429645 | >1.00E-05 | >1.00E-05 | 1.43E-06 | 1.81E-01 | 6.46E-01 | chr3:100053188 | *NIT2* | TSS1500^c^, CGI^b^ shore |
| cg03720762 | >1.00E-05 | >1.00E-05 | 4.82E-06 | 4.17E-01 | 2.93E-01 | chr17:17604184 | *RAI1* | 5'UTR^d^, CGI^b^ shore |
| cg03985718 | 3.61E-06 | 8.54E-01 | 6.53E-05 | 1.99E-01 | 3.45E-01 | chr2:105924245 | *TGFBRAP1* | Body |
| **cg04286697** | **>1.00E-05** | **>1.00E-05** | **1.75E-06** | **1.36E-02** | **1.92E-01** | **chr2:232259623** | ***B3GNT7*** | **TSS1500**^c^**, CGI**^b^ **shore** |
| cg04583842 | >1.00E-05 | >1.00E-05 | 1.33E-06 | 1.46E-01 | 3.12E-01 | chr16:88103117 | *BANP* | Body, CGI^b^ shore |
| **cg04987734** | **4.93E-08** | **4.82E-02** | **1.57E-08** | **8.64E-03** | **4.27E-02** | **chr14:103415873** | ***CDC42BPB*** | **Body** |
| **cg05251389** | **>1.00E-05** | **>1.00E-05** | **9.67E-06** | **3.35E-02** | **4.05E-01** | **chr22:43525330** | ***BIK*** | **3'UTR**^d^ |
| cg05827190 | >1.00E-05 | >1.00E-05 | 6.08E-06 | 8.79E-01 | 7.18E-01 | chr4:681440 | *MFSD7* | Body, CGI^b^ |
| cg06096336 | 8.06E-07 | 3.01E-01 | 2.51E-06 | 4.22E-01 | 5.39E-01 | chr2:231989800 | *PSMD1* | Body |
| **cg07012687** | **3.47E-07** | **1.58E-01** | **4.45E-06** | **8.75E-04** | **3.31E-01** | **chr17:80195180** | ***SLC16A3*** | **Body, CGI**^b^ |
| cg07175797 | >1.00E-05 | >1.00E-05 | 3.92E-07 | 1.36E-01 | 6.60E-01 | chr16:50317656 | NA | IGR^a^ |
| **cg07372520** | **>1.00E-05** | **>1.00E-05** | **6.01E-06** | **5.27E-02** | **2.27E-02** | **chr1:180086434** | **NA** | **IGR**^a^ |
| cg07467649 | >1.00E-05 | >1.00E-05 | 2.75E-06 | 2.26E-01 | 1.14E-01 | chr7:72856462 | *BAZ1B* | 3'UTR^d^ |
| cg07884764 | 5.03E-06 | 9.99E-01 | 1.25E-04 | 6.30E-01 | 9.41E-01 | chr11:64107517 | *CCDC88B* | TSS200^e^, CGI^b^ shelf |
| cg08295111 | 7.87E-06 | 5.76E-01 | 5.64E-04 | 5.89E-01 | 9.56E-01 | chr5:133866097 | *PHF15* | 5'UTR^d^, CGI^b^ shelf |
| cg08631783 | >1.00E-05 | >1.00E-05 | 3.83E-06 | 5.78E-01 | 5.51E-02 | chr15:89418456 | *ACAN* | 3'UTR^d^ |
| **cg08796240** | **7.43E-07** | **2.56E-01** | **1.80E-06** | **5.50E-02** | **2.50E-02** | **chr16:70733832** | ***VAC14*** | **Body** |
| cg09849319 | 1.81E-06 | 4.64E-01 | 1.04E-04 | 8.82E-02 | 9.80E-02 | chr5:1494983 | *LPCAT1* | Body, CGI^b^ |
| cg10401362 | >1.00E-05 | >1.00E-05 | 2.71E-06 | 4.40E-01 | 1.34E-01 | chr7:157185402 | *DNAJB6* | Body |
| cg10829227 | >1.00E-05 | >1.00E-05 | 3.40E-07 | 8.66E-01 | 4.12E-01 | chr19:47200595 | *PRKD2* | Body, CGI^b^ shelf |
| cg11931558 | >1.00E-05 | >1.00E-05 | 5.46E-06 | 5.41E-01 | 9.74E-01 | chr7:11013742 | *PHF14* | 1stExon, CGI^b^ |
| cg12325605 | 9.62E-06 | 9.17E-05 | 5.24E-09 | 2.93E-01 | 6.17E-01 | chr3:56810151 | *ARHGEF3* | Body |
| **cg12526091** | **>1.00E-05** | **>1.00E-05** | **9.00E-07** | **3.85E-02** | **7.75E-01** | **chr12:58245042** | **NA** | **IGR**^a^**, CGI**^b^ **shelf** |
| **cg12728588** | **>1.00E-05** | **>1.00E-05** | **5.43E-07** | **3.64E-02** | **2.51E-01** | **chr1:36025489** | ***NCDN*** | **Body, CGI**^b^ **shore** |
| **cg12764201** | **7.15E-06** | **7.20E-01** | **7.29E-05** | **3.01E-03** | **1.84E-01** | **chr1:10510123** | ***CORT*** | **1stExon** |
| **cg13747876** | **6.32E-06** | **1.04E-01** | **2.93E-06** | **4.74E-02** | **8.10E-01** | **chr17:80195402** | ***SLC16A3*** | **Body, CGI**^b^ |
| cg14012686 | >1.00E-05 | >1.00E-05 | 1.01E-06 | 5.26E-01 | 6.62E-01 | chr2:74785750 | *C2orf65* | 3'UTR^d^, CGI^b^ shelf |
| cg14023999 | >1.00E-05 | >1.00E-05 | 5.99E-08 | 4.82E-01 | 9.02E-01 | chr15:90543224 | NA | IGR^a^, CGI^b^ shore |
| cg16466652 | 4.39E-06 | 3.97E-01 | 1.57E-05 | 2.88E-01 | 3.21E-01 | chr19:6271960 | *MLLT1* | Body, CGI^b^ |
| cg16745930 | 1.34E-06 | 4.01E-01 | 6.26E-06 | 1.88E-01 | 9.36E-01 | chr10:100220809 | *HPSE2* | 3'UTR^d^ |
| cg17237086 | 3.44E-06 | 2.51E-01 | 6.10E-06 | 1.33E-01 | 9.88E-01 | chr22:40814966 | *MKL1* | Body, CGI^b^ |
| cg17743381 | >1.00E-05 | >1.00E-05 | 9.10E-06 | 1.20E-01 | 8.79E-01 | chr1:39024825 | *NA* | IGR^a^ |
| cg17822325 | >1.00E-05 | >1.00E-05 | 2.33E-06 | 2.04E-01 | 3.17E-01 | chr1:31896462 | *SERINC2* | Body |
| cg18030453 | 9.16E-06 | 3.87E-03 | 1.20E-07 | 3.07E-01 | 7.44E-01 | chr3:45506216 | *LARS2* | Body |
| cg19269039 | >1.00E-05 | >1.00E-05 | 3.84E-06 | 1.77E-01 | 4.56E-01 | chr1:111743200 | *DENND2D* | 1stExon, CGI^b^ shelf |
| cg19743103 | >1.00E-05 | >1.00E-05 | 6.08E-06 | 9.27E-01 | 6.56E-01 | chr1:6304220 | *HES3* | TSS200^e^, CGI^b^ shore |
| **cg19769147** | **>1.00E-05** | **>1.00E-05** | **2.09E-06** | **2.63E-02** | **8.08E-02** | **chr14:105860954** | ***PACS2*** | **Body, CGI**^b^ **shelf** |
| cg21098005 | 4.36E-06 | 9.60E-01 | 1.01E-04 | 9.22E-02 | 9.47E-01 | chr20:44538605 | *PLTP* | Body, CGI^b^ shore |
| cg21604136 | >1.00E-05 | >1.00E-05 | 4.93E-06 | 7.86E-01 | 1.49E-01 | chr1:9910137 | *CTNNBIP1* | 3'UTR^d^ |
| cg22069247 | >1.00E-05 | >1.00E-05 | 8.23E-07 | 2.78E-01 | 2.48E-01 | chr2:232393256 | *NMUR1* | Body, CGI^b^ shore |
| cg22365240 | >1.00E-05 | >1.00E-05 | 3.17E-06 | 1.38E-01 | 3.84E-01 | chr2:105374995 | NA | IGR^a^ |
| cg23282441 | 9.69E-06 | 1.77E-01 | 8.63E-06 | 2.19E-01 | 7.32E-01 | chr10:73533927 | *C10orf54* | TSS1500^c^, CGI^b^ shore |
| cg23606718 | >1.00E-05 | >1.00E-05 | 8.37E-06 | 1.09E-01 | 7.65E-01 | chr2:131513927 | *FAM123C* | 5'UTR^d^, CGI^b^ |
| cg24550880 | >1.00E-05 | >1.00E-05 | 5.88E-06 | 3.93E-01 | 7.81E-01 | chr17:79420279 | *BAHCC1* | Body, CGI^b^ |
| cg25392060 | >1.00E-05 | >1.00E-05 | 3.10E-06 | 6.22E-01 | 9.61E-01 | chr8:142297121 | NA | IGR^a^, CGI^b^ shore |
| **cg26610247** | **>1.00E-05** | **>1.00E-05** | **9.70E-06** | **4.74E-02** | **9.54E-01** | **chr8:142297175** | **NA** | **IGR**^a^**, CGI**^b^ **shore** |
| cg27305772 | >1.00E-05 | >1.00E-05 | 6.57E-06 | 3.28E-01 | 8.99E-02 | chr11:65630355 | *MUS81* | Body, CGI^b^ shore |

^a^IGR: Intergenic Region; ^b^CGI: CpG Island; ^c^TSS1500: Probe positioned within 1500 bp region from transcription start site; ^d^UTR:Untranslated region; ^e^TSS200: Probe positioned within 200 bp region from transcription start site
